# Physical and pulmonary capacities of individuals with severe coronavirus disease after hospital discharge: A preliminary cross-sectional study based on cluster analysis

**DOI:** 10.6061/clinics/2021/e3540

**Published:** 2021-11-17

**Authors:** Angelica Castilho Alonso, Paulo Roberto Silva-Santos, Marília Simões Lopes Quintana, Vanderlei Carneiro da Silva, Guilherme Carlos Brech, Lorena Gonçalves Barbosa, José Eduardo Pompeu, Erika Christina Gouveia e Silva, Elizabeth Mendes da Silva, Caroline Gil de Godoy, Julia Maria D’Andréa Greve

**Affiliations:** I Laboratorio de Estudos do Movimento, Instituto de Ortopedia e Traumatologia (IOT), Hospital das Clinicas HCFMUSP, Faculdade de Medicina, Universidade de Sao Paulo, Sao Paulo, SP, BR.; II Programa de Graduacao em Ciencias do Envelhecimento, Universidade Sao Judas Tadeu (USJT), Sao Paulo, SP, BR.

**Keywords:** Coronavirus Disease, Physical Rehabilitation, Functional Physical Performance, Clustering Analysis

## Abstract

**OBJECTIVE:**

This study aimed to analyze the physical and pulmonary capacities of hospitalized patients with severe coronavirus disease and its correlation with the time of hospitalization and complications involved.

**METHODS:**

A total of 54 patients, aged ≥18 years of both sexes, were evaluated 2-4 months after hospital discharge in São Paulo, Brazil. The physical characteristics analyzed were muscle strength, balance, flexibility, and pulmonary function. The K-means cluster algorithm was used to identify patients with similar physical and pulmonary capacities, related to the time of hospitalization.

**RESULTS:**

Two clusters were derived using the K-means algorithm. Patients allocated in cluster 1 had fewer days of hospitalization, intensive care, and intubation than those in cluster 2, which reflected a better physical performance, strength, balance, and pulmonary condition, even 2-4 months after discharge. Days of hospitalization were inversely related to muscle strength, physical performance, and lung function: hand grip D (r=−0.28, *p*=0.04), Short Physical Performance Battery score (r=−0.28, *p*=0.03), and forced vital capacity (r=−0.29, *p*=0.03).

**CONCLUSION:**

Patients with a longer hospitalization time and complications progressed with greater loss of physical and pulmonary capacities.

## INTRODUCTION

Coronavirus disease (COVID-19), a disease caused by severe acute respiratory syndrome coronavirus 2 (SARS-CoV-2), is the greatest challenge facing science today. A world task force from all areas of operation has been established to understand the different nuances related to COVID-19. The disease mainly affects the lungs, causing interstitial pneumonia and severe acute respiratory distress syndrome (1), but has also causes lesions in multiple organs and systems such as cardiovascular, neurological, and muscular systems (2,3).

COVID-19 is a systemic disease with multiple clinical manifestations and may have various sequelae, even in its mildest form. A previous systematic review of post-COVID symptoms stated that 80% of patients may develop at least one persistent symptom after remission of the acute condition. Fatigue and dyspnea are the most prevalent symptoms, in addition to joint pain, hair loss, and attention disorder (4).

In the lungs, COVID-19 leads to injury of the alveolar epithelial and endothelial cells, which proliferates to secondary fibers, causing fibrosis and pulmonary hypertension, with repercussions on the respiratory function of surviving patients (5). Clinical guidelines suggest the follow-up of patients with severe pneumonia because of COVID-19 with pulmonary function tests within 12 weeks of discharge (6).

In the musculoskeletal system, reduction in muscle strength has been observed, especially in patients with prolonged hospitalization. According to Frota et al. (7), this occurs owing to the following three factors: muscle hypoxia, where inadequate perfusion (systemic and peripheral) increases anaerobiosis and blood lactate levels, impairing the muscle function; prolonged immobility, common in hospitalized and/or ICU patients, leading to losses of functional units (sarcomeres) with impairment of postural balance and development of contractures; and the long-term use of corticosteroids and neuromuscular blockers, with polyneuropathies and myopathies.

The recovery process of patients after COVID-19 is not homogeneous, varying according to the severity and time of symptomatology. The repercussions of the disease on long-term functional capacity remain unknown. Physical rehabilitation is necessary for a number of patients post-COVID-19, especially older adults, obese individuals, and patients with other comorbidities. Understanding the functional condition of patients and their limitations, as well as the dynamics of progress after recovering from a severe condition, is important to determine the appropriate rehabilitation process. Several mathematical and statistical models have been proposed to assist in predicting the number of cases and in identifying the characteristics and impact of SARS-CoV-2 infection (2,3,8).

The results of this study, although preliminary, can help identify the characteristics of patients and demands for healthcare services, besides supporting managers and other studies in understanding the epidemiological patterns of the COVID-19 pandemic.

This study aimed to analyze the physical and pulmonary capacities of hospitalized patients with severe COVID-19, based on a cluster analysis, and its correlation with the time of hospitalization and complications involved.

## METHODS

### Study location, study design, and ethical issues

This cross-sectional preliminary study was conducted as part of the results of the project “Avaliação e reabilitação multidisciplinar de pacientes pós-covid- 19: aspectos físicos, neurofisiológicos, imunológicos, microbiológicos, psicológicos e nutricionais” and was approved by the Ethics Committee of the University of São Paulo (CAAE: 39115320.9.0000.0068). The study was conducted at the Laboratory of the Study of Movement (LEM), Hospital das Clínicas of the Faculty of Medicine (HC-FM), University of São Paulo (USP), in partnership with the Graduate Program in Aging Sciences, São Judas Tadeu University (USJT) - SP.

### Participants

A total of 54 male and female COVID-19-confirmed patients aged ≥18 years who were hospitalized at the Central Institute of the HC-FMUSP, discharged from the hospital after 2-4 months, and able to visit the hospital for this research were included in the study. Patients who did not respond to all items in the questionnaire and/or unable to perform all evaluations indicated were excluded.

### Recruitment

Initially, data on demographic characteristics (age and sex) and hospitalization (time of hospitalization and admission to the intensive care unit) were collected from the medical records of patients hospitalized with COVID-19. Those who met the inclusion criteria were invited to participate in the study through a telephone call where the researcher explained clearly and in detail the objectives of the study. In case of agreement, a face-to-face evaluation was scheduled at the LEM HC/FMUSP, where patients were informed about the objectives of the study and signed the informed consent form.

### Instruments

#### Spirometry test

For spirometry, the flow-volume curves were obtained via pneumotachography (preVent, MedGraphics, St. Paul, Minnesota, USA), and the largest volume of the three maneuvers was expressed as the percentage of predicted normal and used for analysis in accordance with the guidelines of the American Thoracic Society-European Respiratory Society (9).

### Muscle strength assessment

Maximal handgrip strength was assessed using the Jamar^®^ dynamometer, with the patient seated and his/her arms parallel to the body, shoulder adduced, elbow flexed at 90°, and forearm and wrist in the neutral position. Three trials were performed for the dominant and non-dominant hands, with a 1-minute interval between trials. The mean value was used for analyses (10).

The stair climbing test was used to measure the time (in seconds) to climb 15 steps (15 cm, height; 30 cm, depth). The participants were requested to perform as quick as possible (11).

The chair stand test was performed using a folding chair without arms, with a seat height of 17 inches (43.2 cm). The chair, with rubber tips on the legs, was placed against a wall to prevent it from moving during the test. Arms were crossed at the wrists and held against the chest. At the signal “go,” the participant rose to a full standing position (body erect and straight) and then returned to the initial seated position. Participants were encouraged to complete as many rounds as possible within a 30-second time limit. The score was determined based on the total number of standing positions executed correctly within 30 seconds (12).

For the core muscle strength and stability test, the participant was positioned in ventral decubitus with forearms and the tip of the metatarsus supported on the ground. The participant was asked to close the soil trunk and maintain this position for as long as possible. The time was measured, and the maximum duration was 90 seconds (13).

### Physical performance assessment

The Short Physical Performance Battery (SPPB) consists of three tests that assess static standing balance (0-4 points); usual walking speed (0-4 points) made in two times of a given round trip; and the muscle strength of the lower limbs, which is evaluated by asking the participant to stand up and sit on the chair five times in a row without the help of the upper limbs (0-4 points). The score ranges from 0 (worst performance) to 12 (best performance) points. The results are rated as follows: 0-3 points, disability or very poor performance; 4-6 points, underperformance; 7-9 points, moderate performance; and 10-12 points, good performance (14).

### Postural balance and functional mobility assessment

The Mini BESTest is a 14-item test. The items are organized in six sections corresponding to the systems that contribute to the maintenance of balance: biomechanical restrictions, stability limits, postural responses, anticipatory postural adjustments, sensory orientation, and dynamic balance during movement and cognition. Each item is scored on a 3-point ordinal scale ranging from 0 to 2 points (best performance) (15).

The Time Up and Go Test (TUGT) measures the time required for an individual to get up from a chair to a standing position, walk 3 m at a normal walking speed, return to the chair, and sit back down. Additionally, the participants performed the TUGT with dual tasks (also known as the “Timed Up and Go [Cognitive]”), in which motor activities are paired with a verbal task, *i.e.*, in this case, individuals were asked to name animals, floors, and colors. The mean value was used for analyses (16).

### Flexibility assessment

The sit and reach flexibility test for the lower limbs and trunk is performed on a 30.5-cm open cube and a scale of 26.0 cm as an extension. The 0 is placed near the evaluated person, while the scale of 26.0 cm is the footrest point. During the test, the participant is barefoot and in the sitting position with his/her feet touching the box and knees extended. With flexed shoulders, extended elbows, and overlapping hands, the participant performs flexion of the trunk in front and attempts to touch the maximum point of the scale with his/her hands. Three attempts are made, and the best mark is considered (17).

### Anthropometric evaluation and body composition

The body mass and height were measured with participants wearing light clothing and without shoes. A digital scale with a stadiometer (Techline TEC^®^, capacity for 136 kg) was used to measure the body mass and height. The body mass index (BMI) was obtained by dividing the weight in kilograms by height in meters squared (kg/m^2^).

### Statistical analysis

R version 4.0.2 was used to perform all analyses, and the open-source library of R was employed. Results of the descriptive analysis of the study population are presented as means and standard deviations. The normality and homogeneity of variances and adherence to the Gauss curve were analyzed using the Shapiro-Wilk test. Spearman’s correlation was used to verify the relationship between physical and pulmonary capacities with hospitalization times, days in the intensive care unit, and intubation. Comparisons by cluster of the values of continuous variables were performed using Student’s t-test and Mann-Whitney test, when appropriate.

### Clustering analysis

The K-means algorithm was used to divide the participants into groups (clusters) based on their similarity, and no hierarchical relationship was observed between the K clusters. Although many clustering algorithms are available according to Vadyala (18), K-means clustering is one of the most popular and widely used algorithms. Each data point is categorized by calculating the distance between the point to each group centroid and then classifying the point closest to it. The centroids are recomputed based on the classified points, and then the process is repeated until the centroids no longer change. In the present study, cluster packages were used for data analysis, while “extra fact” was used to visualize the results. Participants’ data were converted to z-scores (standardized) and input into the algorithm. Standardized data were used to ensure all features have equal influence on the clustering procedure. Two clusters were retained considering the degree of homogeneity in the derived groups and the balance between classes. The interpretability of clusters was examined to confirm the final number of final clusters and if a group was sufficiently large for adequate statistical power, that is, at least 10% of the total sample (19).

## RESULTS

Fifty-four patients (58% were men) were evaluated between December 2020 and June 2021. The mean age was 57.5 (13.4) years, BMI was 29.5 (10.1), and hospital length of stay was 21.4 (16.7) days; 62% were in the intensive care unit, and 61% were intubated.

In the present study, we described the temporal trends in respiratory outcomes after 2-4 months in patients hospitalized because of COVID-19. After hospital discharge, ventilatory abnormalities were assessed using static spirometry. Several patients showed a restrictive response (FVC), obstructive response (FEV1), or mixed airflow changes. Of the 54 patients, 14 (26%) showed restrictive (from mild to severe), 18.5% showed mild, and 7.5% showed mixed airflow changes, of whom three showed moderate airflow changes (75%) and one showed severe airflow changes (25%). Meanwhile, nine patients showed obstructive (16.6%), seven showed mild (12.9%), and two showed mixed airflow changes (3.7%), of whom 50% showed moderate and another 50% showed mild airflow changes.

Table 1 presents the characteristics of COVID-19 survivors hospitalized at HC-FMUSP 2-4 months after hospital discharge.

Table 2 shows the correlation coefficient between days of hospitalization, intensive care and intubation with the other variables.

### Clustering analysis

**Figure 1:**
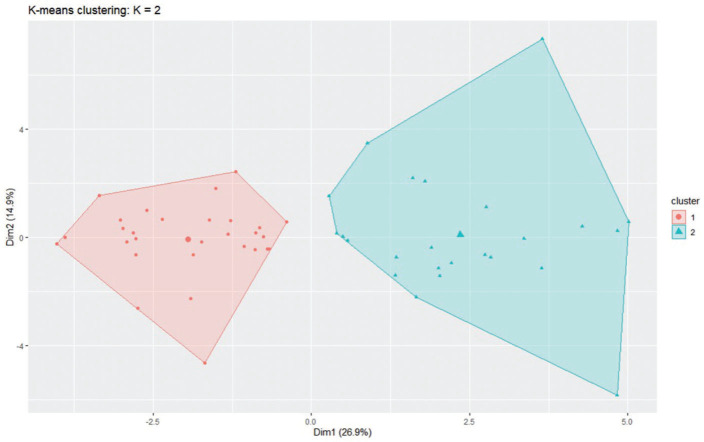
Cluster analysis of COVID-19 survivors with the K-Means algorithm.

Figure 1 shows the result of the cluster analysis with the K-means algorithm. To define the number of K=2, the intra-cluster homogeneity and between-group differences were analyzed. Other subdivisions did not show better cluster homogeneity in addition to resulting in overlap and very small clusters. Therefore, two groups were used to perform clustering. The two dimensions explain 41.8% of the variability of the analyzed data.

**Figure 2:**
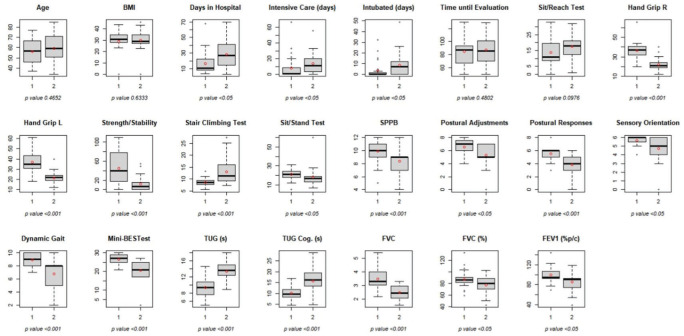
Box plot of tests by Cluster with indication of means (in red circles) and *p*-values for the Student’s t-test. SPPB, short physical performance battery; BMI, body mass index; D, dominant; ND, no dominant; TUG, Timed Up and Go; TUG-COG, Timed Up and Go Cognitive; FEV1, forced expiratory volume in 1 second; FVC, forced vital capacity.

Figure 2 shows the comparison of characteristics related to age, BMI, number of hospitalization days, physical conditions (strength, flexibility, and postural balance), and pulmonary function. Compared with group 2, group 1 had fewer hospitalization, intensive care, and intubation days, which reflected better physical performance, even 2-4 months after discharge. The cluster presented more muscle strength, better balance, and presence of pulmonary conditions.

## DISCUSSION

The main finding of the present study was that the hospitalization time is related to the recovery capacity of these individuals because patients with a longer hospital stay and complications, especially those intubated, required greater immediate rehabilitation.

Prolonged immobilization associated with systemic pathophysiological changes because of the infection affects the functional capacity of patients. Endothelial injury is an important factor of systemic involvement. Two main groups were identified in our sample based on the cluster analysis results. Cluster 1 comprised patients with shorter hospitalization, intensive care, and intubation days; meanwhile, cluster 2 consisted of individuals with longer hospitalization time and complications, in addition to lower strength and balance, and presence of pulmonary conditions based on the results of the evaluations performed 2-4 months after discharge.

Individuals who were hospitalized for longer experienced more severe impairment of muscle strength, possibly owing to the maintenance of the inflammatory process that affected the protein synthesis and caused a reduction in muscle mass (20). Muscle dysfunction in critically ill patients is categorized into early and late stages (21). In the early stage, degradation of muscle proteins and secondary atrophy occur owing to positive regulation in the ubiquitin-proteasome and calpain-caspase pathways, causing autophagy (21). In the late stage, muscle weakness occurs owing to disuse and inability to restore muscle homeostasis and possible pre-existing neuromuscular deficiencies (22). Antigravitational muscles, such as knee extensors and trunk muscles, are also affected by the loss of mechanical load unlike muscles of the upper limbs (23).

According to Mesquita and Gardenghi (24), hospitalized patients lose up to a pound of mass per day; therefore, prolonged immobilization results in severe depletion of muscle mass, strength, and endurance. The loss of lean body mass aggravates their immunological condition, leading to asthenia and higher mortality. Hence, patients are extremely weak upon hospital discharge, with difficulties adapting to the rehabilitation program and performing activities of daily living (personal hygiene, food, and locomotion).

Immobility increases the production of proinflammatory cytokines and reactive oxygen species, enhancing proteolysis and muscle loss (20,25). Up to 40% of muscle strength can be lost within the first week of immobility (23), owing to decreased muscle mass and bone mineral density in addition to impairment in other body systems; these are more evident in individuals with critical disease (22).

Persistent chronic fatigue for weeks and even months is reported by 58% of patients post-COVID 19 and is related to loss of muscle condition. In addition, many patients report joint pain and neuropathic pain, which are associated with fatigue but can also be owing to vascular endothelial lesions or inflammation in other structures caused by the virus (7). Of the 3,762 respondents, >91% reported various kinds of symptoms 35 weeks after the acute phase of the disease. The most frequent symptoms were fatigue, post-exertional malaise, and cognitive dysfunction, but the symptoms’ prevalence varied over time. Approximately 85.9% of the patients experienced relapses triggered by exercise, physical and mental activity, and stress, which may lead to a reduction in work schedule. Hence, the rehabilitation program must be carefully evaluated as well (26).

Impairment in postural balance was also reported, which corroborates with the findings of the study by Lavoura (27); in this study, hospitalized older adults presented with poor balance and had higher incidence of falls after hospital discharge, which led to more hospitalizations. Prolonged hospitalization was an important predictive factor for worst prognosis and incapacity. Inactivity leads to decreased excitation of the neural motor reflex, which may result in a decrease in proprioceptive input to all levels of the central nervous system and/or an increase in the inhibitory activation of interneurons within the backbone, which contributes to a deficit in postural control (28).

After hospital discharge, ventilatory abnormalities were verified using static spirometry at various times (29). Unfortunately, we did not have patients who had the same condition before the COVID-19 pandemic occurred; this would allow a longitudinal assessment of the effect of the disease. However, the spirometry results suggest that at least some of the alterations noted were consequences of COVID-19 after hospitalization (30). Our findings showed restrictive, obstructive, and mixed airflow changes, consistent with other studies (31,32), that required respiratory monitoring after hospital discharge. Several patients had an obstructive pattern while others had restrictive patterns typically indicating a reduction in FEV1 and FVC, and a lower ratio of FEV1/CVF as well. However, the FVC was almost normal because the total capacity of the lungs was not reduced. Therefore, pulmonary rehabilitation is fully justified to mitigate the long-term consequences on the quality of life and functional performance (33).

Although this is a preliminary study, patients with COVID-19 who were discharged from the hospital may have a clear need for rehabilitation, especially those who remained hospitalized and intubated for a longer period. During hospitalization, all patients received treatment, but their physical and pulmonary capacity will remain low and require continuous rehabilitation. In Brazil and Italy (34), timely referral to post-hospitalization rehabilitation centers was difficult, since accessibility was limited as the demand for rehabilitation exceeded the regular capacity of specialized centers. In addition, preventive measures such as lockdowns, restrictions, social distancing, and limited access made it difficult for these patients to receive rehabilitation treatment. Therefore, the patients received slower and/or incomplete post-hospitalization care, which can have consequences such as impairment in independence and autonomy, caregiver burden, increased use of health resources, and mortality.

The limitations of the study are related to the need for face-to-face evaluations in the hospital for debilitated patients. Many of these patients were unable to visit the data collection site (HC-FMUSP). In addition, in accordance with the safety protocols, the study was interrupted a few times, which limited the number of patients to be evaluated. Despite the small sample size, the two clusters identified were very distant from each other, which shows an existing pattern with more homogeneous individuals among themselves and heterogeneous between groups.

The K-means clustering algorithm was used to group patients based on the data on flexibility and muscle strength, physical performance, postural balance, and pulmonary function, indicating that the length of hospital stay may be related to the care that individuals will require to recover after hospital discharge. The results of this study, although preliminary, can help identify the characteristics of patients and demands for healthcare services, besides supporting managers and other studies in understanding the epidemiological patterns of the COVID-19 pandemic.

## CONCLUSION

Longer hospitalization time and complications have negative impacts on physical and pulmonary capacity.

## AUTHOR CONTRIBUTIONS

Alonso AC was responsible for the formal analysis and manuscript writing, review and editing. Silva-Santos PR, Quintana MSL, Barbosa LG, Silva EM and Godoy CG were responsible for investigation and manuscript original draft writing. Silva VC was responsible for theformal analysis. Brech GC and Silva ECG were responsible for supporting investigation and manuscript writing, review and editing. Pompeu JE and Greve JMA were the supervisors responsible for the manuscript writing, review and editing.

## Figures and Tables

**Table 1:** Characterization of coronavirus disease survivors.

	Mean (SD) N=54
Age (years)	57.5 (13.4)
BMI (kg/m^2^)	29.5 (10.1)
Days of hospitalization	21.4 (16.7)
Days of intensive care	10.2 (13.9)
Days intubated	12.9 (10.3)
Time until evaluation (days)	85.8 (20.9)
Flexibility	
Sit and reach test (cm)	15.2 (7.9)
Muscle strength	
Handgrip - R (kg/f)	30.9 (10.3)
Handgrip - L (kg/f)	30.6 (10.9)
Core muscle strength and stability test (s)	32.2 (32.0)
Stair climbing test (s)	10.4 (4.2)
Sit and stand test (1 min)	19.8 (8.1)
SPPB	9.2 (1.8)
Postural balance	
Mini BESTest	
Anticipatory postural adjustments	6.0 (1.5)
Postural responses	4.8 (1.5)
Sensory orientation	5.2 (1.1)
Dynamic gait	8.1 (1.8)
Mini best; total score	24.2 (4.9)
TUG(s)	10.9 (3.0)
TUG-COG(s)	12.3 (4.8)
Pulmonary function test	
FVC	3.0 (0.8)
FVC (%)	83.5 (15.0)
FEV1(% p/c)	93.6 (17.6)

SPPB, short physical performance battery; BMI, body mass index; D, dominant; ND, no dominant; TUG, Timed Up and Go; TUG-COG, Timed Up and Go [Cognitive]; FEV1, forced expiratory volume in 1 second; FVC, forced vital capacity.

**Table 2:** Correlation between hospitalization, intensive care, and intubation days and physical performance/pulmonary function.

	Hospitalization days	Intensive care days	Intubation days
Age (years)	−0.06 (0.65)	−0.03 (0.79)	0.14 (0.30)
BMI (kg/m^2^)	0.04 (0.75)	0.10 (0.47)	0.13 (0.33)
Flexibility			
Sit and reach test (cm)	0.04 (0.77)	−0.01 (0.94)	−0.03 (0.78)
Muscle strength			
Handgrip - D (kg/f)	−0.28 (0.04)*	−0.28 (0.03)*	−0.27 (0.04)*
Handgrip - ND (kg/f)	−0.20 (0.13)	−0.20 (0.13)	−0.19 (0.15)
Core muscle strength and stability test (s)	−0.25 (0.06)	−0.17 (0.21)	−0.11 (0.40)
Stair climbing test (s)	0.38 (0.005)*	0.23 (0.09)	0.13 (0.34)
Sit and Stand test (1 min)	−0.24 (0.08)	−0.13 (0.34)	0.06 (0.62)
Physical performance battery			
SPPB	−0.28 (0.03)*	−0.18 (0.18)	−0.06 (0.66)
Postural balance			
Mini BESTest			
Anticipatory postural adjustments	0.04 (0.74)	0.01 (0.90)	0.04 (0.74)
Postural responses	0.00 (0.99)	−0.09 (0.49)	0.15 (0.28)
Sensory orientation	−0.03 (0.82)	−0.12 (0.38)	−0.10 (0.45)
Dynamic gait	−0.14 (0.30)	−0.08 (0.54)	−0.06 (0.63)
Mini best; total score	−0.09 (0.48)	−0.11 (0.39)	−0.11 (0.42)
TUG (s)	0.24 (0.07)	0.20 (0.13)	0.11 (0.42)
TUG-COG (s)	0.25 (0.06)	0.18 (0.18)	0.07 (0.58)
Pulmonary function test			
FVC	−0.29 (0.03)*	−0.18 (0.18)	0.03 (0.80)
FVC (%)	−0.22 (0.10)	−0.08 (0.52)	−0.26 (0.05)
FEV1 (% p/c)	−0.09 (0.51)	−0.01 (0.93)	−0.14 (0.29)

r Spearman *p*<0.05.

SPPB, short physical performance battery; BMI, body mass index; D, dominant; ND, no dominant; TUG, Timed Up and Go; TUG-COG, Timed Up and Go [Cognitive]; FEV1, forced expiratory volume in 1 second; FVC, forced vital capacity.
